# Relationship between Serum Vitamin D Levels and HDL Cholesterol in Postmenopausal Women from Colombian Caribbean

**DOI:** 10.1155/2018/9638317

**Published:** 2018-12-20

**Authors:** Luz Adriana Sarmiento-Rubiano, José Armando Angarita Ruidiaz, Héctor Fernando Suarez Dávila, Alfonso Suarez Rodríguez, Roberto C. Rebolledo-Cobos, Jimmy E. Becerra

**Affiliations:** ^1^Grupo de Investigación Alimentación y Comportamiento Humano, Nutrition and Diet Program, Metropolitana University, Career 42F No. 75B-18, Barranquilla, Colombia; ^2^Grupo de Investigación Cuidado Crítico y Rehabilitación Funcional, Physiotherapy Program, Metropolitana University, Career 42F No. 75B-18, Barranquilla, Colombia

## Abstract

**Background:**

Previous evidence suggests that metabolic disorders in postmenopausal women could be related with low serum vitamin D levels. For example, vitamin D deficiency has been associated with increased risk factors for cardiovascular disease (CVD), mainly those related with metabolic syndrome.

**Objective:**

To assess the relationship between the serum vitamin D (25-OH-D) levels and the metabolic syndrome markers in postmenopausal women.

**Methods:**

This descriptive and cross-sectional study was conducted in 183 postmenopausal women of four municipalities from Colombian Caribbean. The serum 25-OH-D levels and the anthropometric and biochemical markers were assessed and correlated with metabolic syndrome.

**Results:**

The average value of serum vitamin D (25-OH-D) was 26.34 ± 9.08 ng/mL, and 69.95% of the women had vitamin D levels <30 ng/mL, of which 43.72% were with insufficiency (<30 to >20 ng/mL) and 26.23% with deficiency (<20 ng/mL). Of the evaluated women, the 81.42% seemed to have metabolic syndrome. Through the linear regression, one significant positive association was observed between the HDL cholesterol and the 25-OH-D levels (*P*=0.014).

**Conclusion:**

In the evaluated population in this study, vitamin D deficiency is related with low HDL cholesterol levels.

## 1. Introduction

The vitamin D deficiency has been associated with the increase of risks factors related with MS, mainly the insulin resistance and abdominal obesity [1]. In the last decades and thanks to the scientific advances, it has been possible to establish with solid evidence, for example, that the 25 hydroxycholecalciferol (1,25(OH)2D3), active form of the vitamin D, is an endocrine regulator of the renin angiotensin system, fundamental mechanism in the blood pressure control [2]. Besides, it has been shown that the vitamin D deficiency increases the insulin resistance and reduces its secretion, as well as such deficiency can contribute to the formation of atheromatous plaque demonstrated through several mechanisms [3].

The information that there is so far about the state of vitamin D worldwide is insufficient; however, it has been estimated that more than a billion of people around the world are deficient or insufficient in vitamin D. In United States and Europe, isolated studies have reported deficiencies in more than 50% of the population [4]. In Latin America, the data are not enough, given that, with the exception of Argentina and Mexico, which have a representative sample about the vitamin D levels in the schoolchildren at the national level, the others countries do not have precise data about the state of this micronutrient in their population, but it is estimated that the deficiencies could affect to more than 50% of people [5].

This research work proposes to evaluate the relationship between the serum vitamin D levels and the presence of factors associated to metabolic syndrome among others related with cardiovascular risk in 183 postmenopausal women, who attend to the internal medicine consult to the health institutions of four municipalities from Atlántico department in the Colombian Caribbean.

## 2. Materials and Methods

In this descriptive and cross-sectional study, a convenience sampling of 183 postmenopausal women, who attend habitually to the internal medicine consult to the health institutions (PROMOCOSTA) of four municipalities (Sabana Larga, Baranoa, Malambo, and Santo Tomas) from Atlántico department in the Colombian Caribbean, was conducted.

The samples were collected between the month of September and October of the year 2017. The inclusion criteria were postmenopausal woman (at least one year after the last menstruation period), with ages between 50 and 80 years old, who does not consume replacement hormonal therapy or nutritional supplements or another vitamin D source additional to the diet, who had a stable health status, according to the medical assessment performed by an internal medicine resident under the supervision by an internist, without being exclusion criterion the control of pathologies such as the hypertension and the diabetes through use of medicaments. The medical assessment included the taking of the blood pressure.

### 2.1. Biochemical and Anthropometric Evaluation

The anthropometric assessment that included height in cm, weight in Kg, and abdominal perimeter in cm was performed according to the criteria established in the resolution 2465 of 2016 of the health ministry of Colombia [6], coherent with the procedures established by the World Health Organization (WHO). For the biochemical analyses, the women were cited in morning hours in the health institution, after 12-hour fast for the extraction of whole blood by the vacutainer method in two tubes without anticoagulant. The samples were immediately transported in refrigeration to the Fundación Hospital Universitario Metropolitano (FHUM) laboratories for the determination of glycemia, total cholesterol, high-density cholesterol (HDL), and triglycerides. The determination of serum vitamin D was performed in the ANAMED clinical laboratory, using the LIAISON XL equipment, analyzer of immuno chemiluminescence, and the DIASORIN- LIAISON-25 OH Vitamin D TOTAL kit (Donated for this study by the ANNAR diagnostic company), and the analytical procedure was performed according to the manufacturer's instructions. This kit evaluates the serum 25 hydroxy vitamin D total levels, which corresponds to the serum of the fractions 25-OH-D2 and 25-OH-D3.

### 2.2. Statistical Analysis

An exploratory descriptive analysis of results was carried out to determine the average value of serum 25-OH-D levels in the total population and for each municipality. The absolute frequency and relative frequency of the states of sufficiency, insufficiency, and deficiency of the serum vitamin D were established according to the criteria accepted by ISE (International Society Endocrinology), which define as sufficiency the serum 25-OH-D levels ≥30 ng/mL (75 nmol/L), insufficiency between 21 and 29 ng/mL, and deficiency <20 ng/ml (50 nmol/L).

The presence of metabolic syndrome in postmenopausal women was defined according to the criteria of the Asociación Latinoamericana de Diabetes (ALAD) and the criteria harmonized by the WHO and the International Diabetes Federation (ATP III criteria) according to which the metabolic syndrome is diagnosed in women when there is abdominal obesity with an abdominal perimeter ≥88 cm and two more of the following criteria: high triglycerides >150 mg/dL (or being treated with specific lipid-lowering drug); HDL cholesterol <50 mg/dL (or being treated with effect on HDL); high blood pressure systolic ≥130 mmHg and/or diastolic ≥85 mmHg (or being treated with an antihypertensive), and fasting glycemia >110 mg/dl, glucose intolerance or diabetes. The associated variables to MS along with the total cholesterol, LDL cholesterol, and the presence of overweight and obesity also considered as factors of CVR were included in the correlation analyses with the vitamin D serum value and the ANOVA test according to the vitamin D status, using the Statgraphics Plus statistical program.

### 2.3. Ethical Aspects

This study was carried out with previous authorization of ethics committee of the Metropolitana University. The participants signed the informed consent according to the ethical norms stipulated in the Resolution 8430 of 1993 of the Colombia Ministry of Health and the Helsinki declaration. The published information does not contain sensitive data of the participants, and there was written authorization from participating health entity.

## 3. Results

In this descriptive cross-sectional study, 183 postmenopausal women were included with ages between the 50 and 80 years old with an average age of 64.21 ± 6.12 years old, who attend habitually to the internal medicine consult in the Promocosta IPS from the municipalities of Sabana Larga, Baranoa, Malambo, and Santo Tomas. The average values of the anthropometric, biochemical, age, blood pressure, and serum vitamin D levels variables of the population are shown in Table 1, as well as the comparison between the values of the mean between the municipalities (ANOVA test with statistical significance when *P* ≤ 0.05). Serum determination of the vitamin D levels shows mean values in the total population of 26.34 ± 9.08 ng/ml, corresponding to a rank between 12.5 and 64.5 ng/ml, being statistically significantly lower in the municipalities of Baranoa and Sabana Larga in relation to what was found in Malambo and Santo Tomás (Table 1).

The anthropometric assessment allowed to classify the population analyzed according to the body mass index (BMI): eutrophic women (normal BMI; 18.5–24.9), overweight women (BMI; 25–29.9), and obese women (BMI >30), without showing significant differences between municipalities (chi-square test; *P* > 0.05) (Table 2). The frequency of deficiency (25-OH-D <20 ng/mL), insufficiency (between 21 and 29 ng/mL), and sufficiency (>30 ng/mL) of vitamin D in the total population and for each municipality was determined (Table 2). Baranoa and Sabana Larga municipalities had the frequencies greater of women with deficiency and insufficiency of vitamin D than those found in Santo Tomas and Malambo (*P* ≤ 0.001; chi-square test).

The presence of metabolic syndrome (MS) was determined according to the ATP III criteria in 149 of the 183 evaluated women (81.42%), being Baranoa the municipality with the highest frequency of women with MS (88.37%) and Sabana Larga was the municipality which shows lower frequency (67.50%) (Table 2). Besides the abdominal perimeter that is greater than 88 cm, risks factors associated to MS present in the women were the following: (i) arterial hypertension present in 147 (98.65%) of the diagnosed women with MS; of these, 141 had previous diagnosis and treatment with antihypertensive and 6 without previous diagnosis but, together with 56 those diagnosed as diabetic, had high blood pressure levels at the time of the assessment; (ii) the fasting glycemia criterion higher than 110 mg/dL, glucose intolerance or diabetes, was present in 63 (42.28%) of the women with MS, 39 had previous diagnosis of diabetes and treatment with oral hypoglycemic agents, and 8 of these, along with 24 other women not diagnosed as diabetic, had high basal glycemia levels at the time of assessment; (iii) high triglyceride values were found in 112 (75.16%) of women with MS (range 150.6–370 mg/dL); and (iv) 84 women (56.37%) had HDL values <50 mg/dL (range 24.5–49.8 mg/dL).

The existing relationship between serum vitamin D levels and each variable was evaluated through an analysis of lineal regression. Only one positive linear correlation was found between the vitamin D values and the HDL cholesterol levels (*R*^2^ = 3.27%, *P*=0.014) (data not shown). These results were corroborated when each of the variables was compared with the vitamin D status through an ANOVA test. Statically significant differences (*P*=0.002) were found only with the HDL variable, showing the lowest values of this variable only in the population that had deficient and insufficient vitamin D values (Table 3).

In each municipality, the relationship between the presence or absence of MS and the sufficiency, deficiency, or insufficiency of vitamin D was established, without observing any statistically significant correlations in any municipality or in the total population evaluated (*P* > 0.05 Chi^2^ test). However, it is noted that in the municipality of Baranoa (*P*=0.053), 100% of the women with MS have vitamin D lower than 30 ng/ml. It is worth highlighting also that this municipality presented the highest levels of deficiency or insufficiency in the evaluated population (97.67%), with 42 of the 43 women in a deficiency or insufficiency condition and only one woman with sufficiency of vitamin D without presenting MS; besides, Baranoa was the municipality with the highest percentage of MS with 88.37% (38 women with MS of the 43 evaluated). In Malambo, Sabana Larga and Santo Tomas of the women with MS and who had vitamin D levels lowest to 30 ng/ml were 47.36%, 96.29%, and 52.17% respectively.

## 4. Discussion

In this study was found that of 183 postmenopausal women evaluated, 69.95% had serum levels of 25-OH-D lower than 30 ng/ml, with 26.23% of the population in the condition of deficiency (<20 ng/ml of 25-OH-D), and the results are consistent with those of the Colombian experts' study recently published, who based on the WHO reports calculate that two out of three postmenopausal women have vitamin D deficiency [7]. In the Survey of the Nutritional Situation in Colombia (ENSIN) in its 2015 version, an evaluation of the serum vitamin D levels was performed in the Colombian population; however, the results have not yet been published, finding only a few current references of studies at national level on the subject.

In the city of Medellín Colombia, between the years 2004 to 2006, Hormanza and collaborators evaluated the serum vitamin D levels through the ELISA technique in 113 women aged between 20 and 75 years old approximately, finding that 76% of them had vitamin D values lower than 10 ng/ml, being the more deficient menopausal women than the no menopausal or postmenopausal women [8]. In the city of Villavicencio of this same country, 60.37% of 106 postmenopausal women evaluated had levels of deficiency or insufficiency of vitamin D [9]. A study published in 2010, which evaluated 205 postmenopausal women diagnosed with osteoporosis and osteopenia in the city of Medellín, showed that 55.1% had insufficient levels of vitamin D and the 16.6% of this population was deficiency [10].

The previous findings, along with what was found in this study, although with important methodological and analytical differences, show that in Colombia, the vitamin D deficiency in postmenopausal women constitutes an important health problem, situation that possibly extends to other population groups accordingly reported by a study of the Metropolitan Area of Barranquilla, in the department of Atlantic, that found insufficient levels of vitamin D in 46.38% of the children under 10 years old and deficient in the 3.05% [11]. For other Latin America countries, inadequate vitamin D values could be present in about 67% of the population according to the collected data from Argentina, Brazil, Chile, and Mexico [12].

The relationship of the vitamin D with the cardiovascular risk and presence of MS has been previously reported in other studies. In the United States of America, for example, more than 8000 citizen of both sexes were evaluated, finding an inverse relationship between the serum vitamin D values and the abdominal circumference, hypertriglyceridemia, and hyperglycemia [13]. In 3577 adolescents included in the NHENES III study was found a direct association between low levels of serum vitamin D and high blood pressure, hyperglycemia, and MS [14]. In this study, serum vitamin D values were related with each one of the variables associated with MS or CVR as total cholesterol and LDL. The results of the linear regression showed a relationship only between the HDL cholesterol and vitamin D levels, which was confirmed when the analysis was performed comparing the vitamin D status and the variables through an ANOVA test.

Although the molecular mechanism by which vitamin D is related with serum levels of HDL cholesterol are not fully elucidated, studies such as that conducted in Finland with 909 men between 45 and 70 years old have reported a direct relationship between low levels of HDL cholesterol and deficiency of vitamin D mainly of the active form 1,25-OH-D [15]. A study with 4274 English children showed that the high levels of 25-OH-D are associated with cardioprotective levels of HDL, apoprotein A1, and adiponectin [16], a relation that was confirmed in an experimental study with Iranian children with ages between 10 to 14 years old, in whom the supplementation of vitamin D showed a significant positive impact on the serum HDL cholesterol levels in relation with the control group [17]. It is necessary to study more deeply the mechanisms by which the vitamin D improves the HDL cholesterol levels, being this a key mechanism to the prevention of cardiovascular risk.

## 5. Conclusions

The results of this study allowed to determine that there is a high frequency in the serum vitamin D deficiency/insufficiency of postmenopausal women from Colombian Caribbean, as well as its relationship with factors associated to metabolic syndrome (MS) and cardiovascular risk (CVR) was determined, finding only a significant positive association between the vitamin D deficiency and low values of HDL cholesterol. These results must alert at the Colombian health system to increase the measures of promotion and prevention where it includes the serum vitamin D evaluation in the population and its inclusion in supplementation programs, in order to decrease such deficiencies and the risk factors that are predisposing to cardiovascular disease and metabolic syndrome.

## Figures and Tables

**Table 1:** Anthropometrics and biochemical characteristics and blood pressure of the studied women population.

Variables	Total	Population by municipalities				*P* ^4^ value
	Population	Baranoa	Malambo	Sabana Larga	Santo Tomas	
Population (*n*)	183	43	44	40	56
Age (años)	64.21 ± 6.12	64.53 ± 5.51	63.68 ± 5.39	65.30 ± 6.40	63.59 ± 6.90	0.517
Height (cm)	156.46 ± 7.76	155.49 ± 5.65^ab^	154.82 ± 11.51^a^	156.78 ± 6.51^ab^	158.27 ± 5.93^b^	0.124
Weight (kg)	72.79 ± 12.64	68.74 ± 8.49^a^	76.93 ± 11.77^b^	73.21 ± 14.93^ab^	72.34 ± 13.45^ab^	**0.024** ^*∗*^
BMI	29.86 ± 5.86	28.46 ± 3.42^a^	32.71 ± 8.28^b^	29.65 ± 4.85^a^	28.85 ± 5.02^a^	**0.001** ^*∗*^
Ab. perimeter (cm)	100.96 ± 9.43	100.70 ± 8.78^ab^	102.66 ± 9.10^a^	98.23 ± 11.21^b^	101.77 ± 8.55^ab^	0.155
Glycemia (mg/dL)	92.04 ± 28.93	82.95 ± 22.10^a^	88.72 ± 24.51^ab^	94.17 ± 35.88^ab^	100.03 ± 29.53^b^	**0.024** ^*∗*^
Cholesterol (mg/dL)	183.74 ± 48.55	170.98 ± 40.90^a^	199.62 ± 58.29^b^	186.08 ± 34.88^ab^	179.39 ± 51.48^a^	**0.040** ^*∗*^
Triglycerides (mg/dL)	182.18 ± 70.89	192.23 ± 78.89	184.72 ± 77.02	164.10 ± 63.58	185.39 ± 63.54	0.305
HDL (mg/dL)	51.67 ± 11.53	43.59 ± 9.53^a^	53.17 ± 11.10^bc^	51.48 ± 7.71^b^	56.85 ± 12.31^c^	**0.000** ^*∗*^
LDL (mg/dL)	95.63 ± 40.61	88.94 ± 33.10^ac^	109.51 ± 48.15^b^	101.78 ± 29.86^ab^	85.47 ± 43.07^c^	**0.012** ^*∗*^
BP-systolic	125.73 ± 12.22	125.95 ± 10.16^ab^	124.64 ± 12.45^ab^	122.75 ± 7.16^a^	128.55 ± 15.56^b^	0.124
BP-diastolic	78.14 ± 7.33	76.84 ± 5.13^ab^	75.66 ± 8.26^a^	78.80 ± 5.87^bc^	80.61 ± 8.19^c^	**0.003** ^*∗*^
Vitamin D (ng/ml)	26.33 ± 9.07	19.96 ± 4.14^a^	32.49 ± 10.07^b^	20.61 ± 4.40^a^	30.47 ± 7.93^b^	**0.000** ^*∗*^

The average values ± standard deviation of the age, height, weight, BMI, Ab. perimeter, basal glycemia, total cholesterol, triglycerides, HDL cholesterol, LDL cholesterol, blood pressure, and serum vitamin D levels of the total population and by municipalities are shown. *P* is the value of statistical significance of the comparison (ANOVA test) between municipalities that is representative if it is less than 0.05^*∗*^. The averages in the same row that does not share the same letter of superscript are different. BMI = body mass index; Ab. perimeter = abdominal perimeter; BP = blood pressure in mmHg.

**Table 2:** Categorization of the studied population according to the anthropometric variables, serum vitamin D levels, and presence of MS.

		Total	Baranoa	Malambo	Sabana Larga	Santo Tomas
Nutritional diagnosis	Normal (%)	18.58	16.28	11.36	12.50	30.36
	Overweight (%)	40.98	53.49	34.09	47.50	32.14
	Obesity (%)	40.44	30.23	54.55	40.00	37.50

Vitamin D status	Sufficient (%)	30.05	2.33	52.27	5.00	51.79
	Insufficient (%)	43.72	46.51	38.64	55.00	37.50
	Deficient (%)	26.23	51.16	9.09	40.00	10.71
	Def. + ins. (%)	69.94	97.67	47.72	95.00	48.21

MS (%)		81.42	88.37	86.36	67.50	82.14

The percentage in the total population and by municipality for each condition according to the classification in relation to the variables is shown. Nutritional diagnosis was done by anthropometry according to BMI (normal <25, overweight >25<30, and obesity >30). Vitamin D status was considered according to the serum values of 25-OH-D (deficient <20 ng/mL, insufficient 21–29 ng/mL, and sufficient >30 ng/mL).

**Table 3:** Average values of the anthropometrics and biochemicals variables according to status of the vitamin D in the total population.

Variables	Total population	Status of vitamin D			*P* ^3^ value
		Deficient <20 ng/mL	Insufficient 21–29 ng/mL	Sufficient >30 ng/mL	
Population (%)	183	48 (26.22%)	80 (43.71%)	55 (30.22%)	
Age (years)	64.21 ± 6.12	64.85 ± 5.28	63.68 ± 5.93	64.42 ± 7.06	0.549
Weight (kg)	72.79 ± 12.64	71.83 ± 11.84	73.83 ± 12.60	72.11 ± 13.48	0.616
BMI	29.86 ± 5.86	29.70 ± 7.28	30.10 ± 4.40	29.66 ± 6.41	0.889
Ab. perimeter (cm)	100.96 ± 9.43	101.67 ± 8.35	99.95 ± 10.37	101.80 ± 8.89	0.446
Glycemia (mg/dL)	92.04 ± 28.93	94.25 ± 29.85	92.13 ± 32.66	89.91 ± 21.61	0.754
Cholesterol (mg/dL)	183.74 ± 48.55	190.78 ± 62.63	178.03 ± 35.53	185.91 ± 50.84	0.330
Triglycerides (mg/dL)	182.18 ± 70.89	198.72 ± 78.23	174.67 ± 69.38	178.68 ± 65.03	0.161
HDL (mg/dL)	51.67 ± 11.53	48.05 ± 11.78^a^	51.08 ± 9.72^a^	55.69 ± 12.66^b^	**0**.**002**^*∗*^
LDL (mg/dL)	95.63 ± 40.61	102.98 ± 48.08	92.01 ± 31.94	94.48 ± 44.56	0.325
BP-systolic	125.73 ± 12.22	124.81 ± 9.19	125.80 ± 11.57	126.44 ± 15.25	0.797
BP-diastolic	78.14 ± 7.33	77.46 ± 4.75	78.41 ± 7.33	78.33 ± 9.06	0.757
Vitamin D (ng/ml)	26.33 ± 9.07	17.06 ± 2.07	24.10 ± 3.05	37.66 ± 6.76	

The average values ± standard deviation of the age, weight, BMI, Ab. perimeter, basal glycemia, total cholesterol, triglycerides, HDL cholesterol, LDL cholesterol, and blood pressure according to the serum vitamin D levels of the total population are shown. P is the value of statistical significance of the comparison (ANOVA test) between vitamin D levels (deficient, insufficient, and sufficient) that is representative if it is less than 0.05^*∗*^. The averages in the same row that do not share the same letter of superscript are different. BMI = body mass index; Ab. perimeter = abdominal perimeter; BP = blood pressure in mmHg.

## Data Availability

The data used to support the findings of this study are available from the corresponding author upon request.

## References

[1] Ford E. S., Ajani U. A., McGuire L. C., Liu S. (2005). Concentrations of serum vitamin D and the metabolic syndrome among U.S. Adults. *Diabetes Care*.

[2] Wang T. J., Pencina M. J., Booth S. L. (2008). Vitamin D deficiency and risk of cardiovascular disease. *Circulation*.

[3] Boucher B. J. (2007). Inadequate vitamin D status: does it contribute to the disorders comprising syndrome “X”?. *British Journal of Nutrition*.

[4] Holick M. F., Chen T. C. (2008). Vitamin D deficiency: a worldwide problem with health consequences. *The American Journal of Clinical Nutrition*.

[5] Brito A., Cori H., Olivares M., Mujica M. F., Cediel G., de Romaña D. L. (2013). Less than adequate vitamin D status and intake in Latin America and the Caribbean: a problem of unknown magnitude. *Food and Nutrition Bulletin*.

[6] Zanuy M., Carranza F. (2007). Metabolismo, fuentes endógenas y exógenas de vitamina D. *Revista Española de Enfermedades Metabólicas Óseas*.

[7] Martini L. A., Wood R. J. (2006). Vitamin D status and the metabolic syndrome. *Nutrition Reviews*.

[8] Vásquez-Awad D., Cano-Gutiérrez C. A., Gómez-Ortiz A. (2017). Vitamina D. Consenso colombiano de expertosd. *Medicina*.

[9] Hormaza M. P., Cuesta D., Martínez L. M. (2011). Niveles séricos de 25 hidroxivitamina D en mujeres no menopáusicas, menopáusicas y posmenopáusicas. *Revista Colombiana de Obstetricia y Ginecologia*.

[10] Molina J. F., Molina J., Escobar J. A., Betancur J. F., Giraldo A. (2011). Niveles de 25 hidroxivitamina D y su correlación clínica con diferentes variables metabólicas y cardiovasculares en una población de mujeres posmenopáusicas. *Acta Médica Colombiana*.

[11] Acosta-Bendek B. M., Sánchez-Majana L. P., Sarmiento-Rubiano L. A. (2017). Estado de la 25-hidroxivitamina D séricaen niños sanos menores de 10 añosdel área metropolitana de Barranquilla. *Salud Pública de México*.

[12] Mithal D. A., Bonjour (2009). On behalf of the IOF committee of scientific advisors (CSA) nutrition working group. Global vitamin D status and determinants of hypovitaminosis D (2009) osteoporosis international. *Osteoporosis International*.

[13] Lu L., Yu Z., Pan A. (2009). Plasma 25-hydroxyvitamin D concentration and metabolic syndrome among middle-aged and elderly Chinese individuals. *Diabetes Care*.

[14] Reis J. P., von Mühlen D., Miller E. R., Michos E. D., Appel L. J. (2009). Vitamin D status and cardiometabolic risk factors in the United States adolescent population. *Pediatrics*.

[15] arhapä P., Pihlajamäki J., Pörsti I. (2010). Diverse associations of 25-hydroxyvitamin D and 1,25-dihydroxy-vitamin D with dyslipidaemias. *Journal of Internal Medicine*.

[16] Williams D. M., Fraser A., Sayers A. (2012). Associations of 25-hydroxyvitamin D2and D3with cardiovascular risk factors in childhood: cross-sectional findings from the avon longitudinal study of parents and children. *Journal of Clinical Endocrinology and Metabolism*.

[17] Tavakoli F., Namakin K., Zardast M. (2016). Vitamin D supplementation and high-density lipoprotein cholesterol: a study in healthy school children. *Iranian Journal of Pediatrics*.

